# Finite Element Method-Based Skid Resistance Simulation Using In-Situ 3D Pavement Surface Texture and Friction Data

**DOI:** 10.3390/ma12233821

**Published:** 2019-11-21

**Authors:** Yi Peng, Joshua Qiang Li, You Zhan, Kelvin C. P. Wang, Guangwei Yang

**Affiliations:** 1 School of Civil Engineering, Southwest Jiaotong University, Chengdu 610031, China; py.peng@outlook.com (Y.P.); zhanyou@swjtu.edu.cn (Y.Z.); 2 School of Civil & Environmental Engineering, Oklahoma State University, Stillwater, OK 74078, USA; kelvin.wang@okstate.edu (K.C.P.W.); guangwy@okstate.edu (G.Y.)

**Keywords:** skid resistance, pavement texture, finite element method, binary search back-calculation approach, principal component analysis regression

## Abstract

Skid resistance is an important surface characteristic that influences roadway safety. Various studies have been performed to understand the interaction between pavement and tires through numerical simulation for skid resistance prediction. However, the friction parameters required for simulation inputs are generally determined by objective assumptions. This paper develops a finite element method (FEM)-based skid resistance simulation framework using in-situ 3D pavement surface texture and skid resistance data. A 3D areal pavement model is reconstructed from high resolution asphalt pavement surface texture data. The exponential decay friction model is implemented in the simulation and the interface friction parameters required for the simulation are determined using the binary search back-calculation approach based on a trial process with the desired level of differences between simulated and observed skid numbers. To understand the influence of texture characteristics on interface friction parameters, the high-resolution 3D texture data is separated into macro- and micro-scales through Butterworth filtering and various areal texture indicators are calculated at both levels. Principal component analysis (PCA) regression analysis is conducted to quantify the relationship between various texture characteristics and the interface friction parameters. The results from this study can be used to better prepare the inputs of friction parameters for FEM simulation.

## 1. Introduction

The risk of traffic accidents may rise significantly when the skid resistance is lower than a certain threshold [1]. The skid resistance is measured as a resistive drag force, generally using the locked-wheel, dynamic friction tester or grip tester with standard testing tires or rubber sliders [2]. The pavement interface friction is affected by many factors, such as vehicle factors (load, speed, slip ratio), rubber properties, asphalt pavement factors (aggregate shape, roughness, micro- and macro-texture), and weather conditions (temperature and contamination) [3,4,5].

Many researchers have contributed to monitoring and predicting pavement friction in the past decades [6]. Macro- and micro- pavement textures have been found to contribute significantly to surface friction and various relationships have been developed [3,7,8,9]. With advances in noncontact three-dimensional (3D) measurement technologies and developments in high performance computers, wavelet analysis, the Hilbert–Huang transform, fractal analysis, power spectra density, and Persson’s model have been used to characterize pavement macrotexture attributes and correlate them with friction performance [10,11,12,13,14,15]. Besides the traditional texture parameters, such as the Mean Profile Depth (MPD), Li et al. [16] selected an array of three-dimensional (3D) areal texture parameters to predict surface friction at various speeds.

In addition, the analytical method is another way to mathematically address the interface friction [17,18,19,20,21]. Brush tire model and vibration-based methods have been adopted to estimate the friction coefficient in the previous researches [22]. Several studies [23,24,25] concluded that the rubber sliding friction coefficient was the most influential factor in pavement interaction skid resistance, which increased with sliding velocity until a threshold value was reached at a certain speed, and then subsequently declined with the speed. Dorsch et al. [26] found that nonlinear relationships existed among the rubber asphalt pavement interface friction coefficient, sliding speed, and temperature. Recently, several researchers [27,28,29,30] have proposed the finite element method (FEM) tire-pavement interaction model and analyzed the tire-pavement contact stress distributions at various conditions. Fwa and Ong [31] presented the back-calculation method to determine the interface friction parameters from the skid resistance FEM simulation model. Wang and Al-Qadi, [32] and Zhou et al. [33] investigated the influence of rubber asphalt pavement interface friction and tire maneuvering on tire asphalt pavement contact stresses and concluded that the exponential decay friction model proposed by Oden and Martins [34] was reasonable to predict the tire asphalt pavement interaction. Researchers from Delft University of Technology (TU Delft) [35,36,37,38] developed a temperature depended skid resistance simulation model considering actual asphalt pavement surface morphologies from X-ray scanning images. The relationship between skid resistance and traditional texture parameters such as MPD and mean texture depth (MTD) was discussed. However, despite extensive advancements in this area, few studies have integrated in-situ high resolution 3D areal texture surface data sets for rubber pavement interface friction simulation. As a result, an appropriate rubber asphalt pavement interaction model with rich pavement texture characteristics is needed to capture the realistic rubber asphalt pavement interface friction behavior.

## 2. Objective

**Figure 1:**
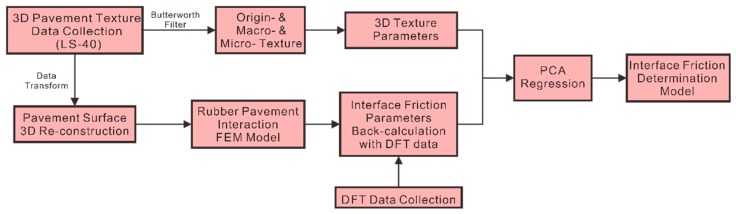
Flowchart to determine interface friction.

The objective of this paper is to develop a rubber pavement interaction simulation framework to determine the interface friction of pavement surfaces using in-situ 3D pavement surface texture and skid resistance value. As illustrated in Figure 1, the framework is composed of the following components:

- Collecting in-situ high-resolution 3D pavement surface texture and skid resistance measured by the dynamic friction tester (DFT);
- Characterizing pavement texture attributes at both macro- and micro-scale with 3D areal texture parameters; the Butterworth filter is applied to separate the texture data into two scales;
- Proposing a re-construction method to establish 3D areal pavement surfaces as inputs for the FEM numerical simulation below;
- Implementing the FEM-based rubber pavement interaction model to simulate the DFT measurements;
- Computing the rubber pavement interface friction using the DFT data sets according to the binary search back-calculation method;
- Determining the relationships between rubber pavement interface friction and the 3D areal pavement texture parameters using principal component analysis PCA regression.

## 3. Field Data Collection

**Figure 2:**
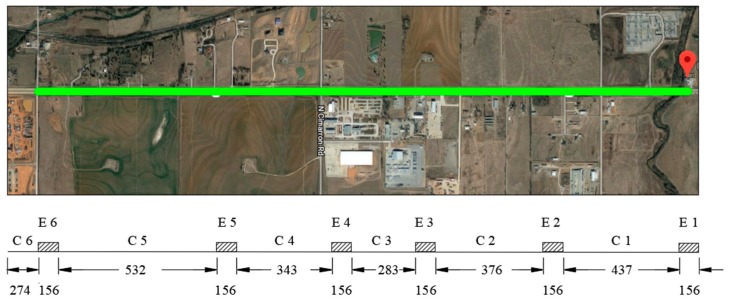
LTPP SPS-10 Sites in Oklahoma (unit: m) (Source: Google Map). Note: E1–E6 the experimental treatment sections; C1–C6 the control sections. Note: E1–E6 the experimental treatment sections; C1–C6 the control sections.

The field-testing bed of this study is the long-term pavement performance (LTPP) Specific Pavement Study 10 (SPS-10) sites, which were constructed by the Oklahoma Department of Transportation (DOT) on Highway 66 in Yukon in November 2015. The experimental matrix, to evaluate the short- and long-term performance of the asphalt mixtures, includes one hot mix asphalt (HMA) and two warm-mix asphalt (WMA) experimental treatment sections, where a foaming process and a chemical additive with 10% to 25% reclaimed asphalt pavement (RAP) and reclaimed asphalt shingle (RAS) content are required per LTPP [39]. Under the SPS-10 experiment treatment, six LTPP SPS-10 experimental treatment sections and six control sections were constructed in Oklahoma. The site location and the corresponding length for each section are shown in Figure 2. The detailed mixture design for the sites is presented by Table 1. High resolution pavement 3D surface texture and DFT skid resistance data sets were collected in the field on the same day in January 2017. The average ambient temperature during the testing is 16 °C.

**Figure 3:**
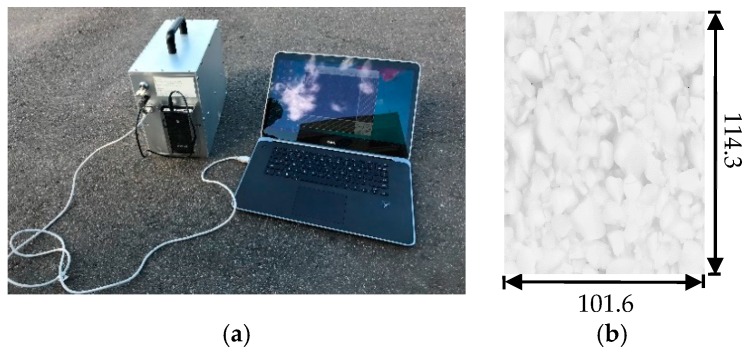
LS-40 pavement surface texture scanner and example of the data: (**a**) LS-40 PorTable 3D Surface Analyzer (HyMIT Measurement Instrument Technology, Austin, TX, USA); (**b**) Example 3D Range Image (unit: mm)/.

In particular, two instruments are used in the field for texture and skid resistance data collection. 3D pavement surface texture data was obtained utilizing the LS-40 porTable 3D surface analyzer, as shown in Figure 3. This analyzer scans an area of 101.6 mm by 114.3 mm with the height resolution (z) of 0.01 mm and lateral resolution (x,y) of 0.05 mm [40]. The high-resolution 3D texture data acquired from LS-40 includes both macro- and micro-level texture information of the scanned surfaces. Each scan has 2048 by 2448 cloud points.

**Figure 4:**
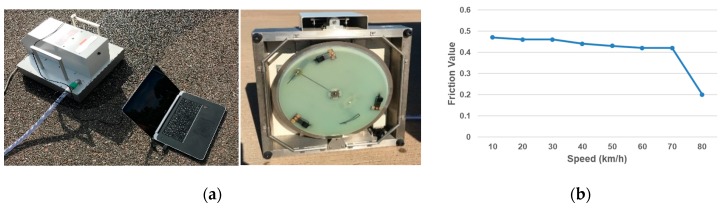
DFT and example data set: (**a**) Dynamic Friction Tester (DFT) (Nippo Sangyo Co., LTD., Tokyo, Japan); (**b**) Example DFT Friction Data.

Skid resistance data was collected using DFT, which measures pavement surface frictional properties at various speeds [41]. DFT is widely used for skid resistance because it is repeatable and reproducible with controlled operating procedures or ambient factors [42]. It consists of a horizontal spinning disk mounted with three spring-loaded rubber sliders, as shown in Figure 4. Water spray in front of the rubber sliders to form 1 mm water film thickness and a constant vertical load is applied on the slider, while the disk spins on the test surface. The torque signal is monitored constantly while the velocity of the spinning disk decreases due to the friction between the rubber sliders and the test surface, thus the pavement surface skid resistance data is derived.

Within each LTPP SPS-10 section, three pairs of LS-40 3D surface texture data and DFT skid resistance data were obtained at 100-ft intervals, starting at the beginning of each section. On the mainline control section after each experimental treatment section, an additional three pairs of pavement texture and skid resistance measurements were conducted at 300-ft intervals. Therefore, 36 pairs of 3D pavement texture and skid resistance data measurements were obtained for each data collection.

## 4. Interface Friction Model

Interface friction occurs at the contact surface between the pavement and the tire, resulting from adhesion and hysteresis. The adhesion is related to interface shear strength while the hysteresis is the result of damping losses and energy dissipation of the rubber arising from the pavement surface asperities [32].

**Figure 5:**
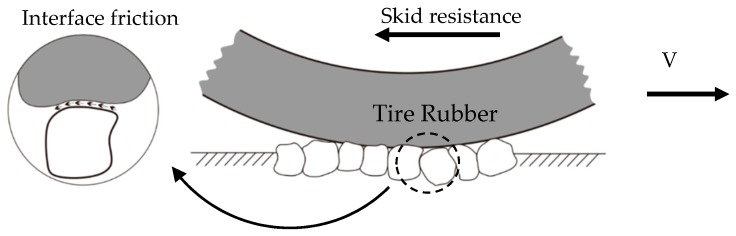
Skid resistance and interface friction.

**Figure 6:**
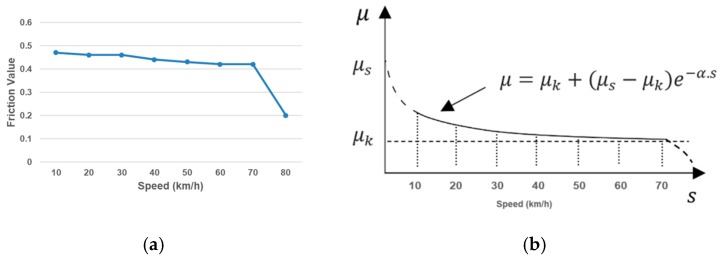
Comparison of DFT friction data with the exponential decay friction model: (**a**) DFT Friction Data; (**b**) Exponential Decay Friction Model (Oden and Martins, 1985).

Many studies have concluded that the skid resistance performance mainly depends on the rubber pavement interface friction [2,3], as illustrated in Figure 5. To describe the interface friction property, the exponential decay friction model proposed by Oden and Martins [34] is generally used. One example DFT testing is illustrated in Figure 6, which has demonstrated a similar trend as that of the exponential decay friction model. The exponential decay function is shown in Equation (1):

(1) $$\mu =\mu_{k}+\left(\mu_{s}-\mu_{k}\right)e^{-\alpha .s}$$

where *μ_s_* is the static friction coefficient, *μ_k_* is the kinetic friction coefficient, *α* is a user-defined decay coefficient, and *s* is the sliding velocity. In this study, the rubber pavement interaction is lubricated with water films during the simulation [43]. The influence of water on the interface friction is reflected in the three friction input parameters in the exponential decay function: *μ_s_* the static friction coefficient, *μ_k_* the kinetic friction coefficient, and α the decay coefficient.

## 5. Reconstruction of 3D Pavement Texture Surface

To accurately illustrate the rubber pavement contact mechanism, many pavement surface models have been established based on sine patterns [44], X-ray tomography [36], simplified porous pavement surface [45], hemispheric roughness surface [46] and other forms [47,48,49,50], to reveal the transient dynamic performance of rubber when the tire traversed over a pavement segment [44].

**Figure 7:**
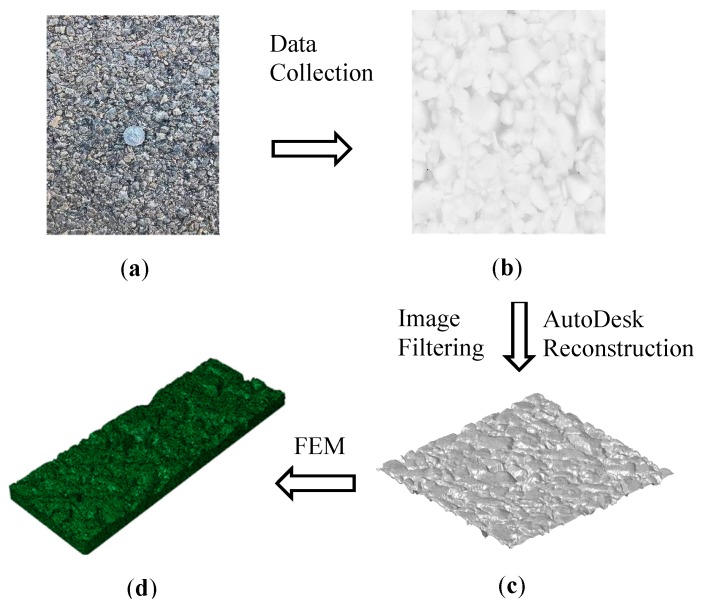
Reconstruction of 3D texture surface for FEM simulation: (**a**) Field Data; (**b**) LS-40 Range Data; (**c**) Re-constructed Data; (**d**) Pavement FEM Model.

In this paper, the areal pavement surface model is reconstructed based on the high-resolution 3D LS-40 texture data sets. Figure 7 shows the reconstruction procedure of the 3D texture surface for FEM simulation mesh using field data. Pavement surface texture images are obtained using LS-40 (HyMIT Measurement Instrument Technology, Austin, TX, USA) from the field and saved as 2048 × 2448 16-bit range data. Speckle noises can exist in the LS-40 pavement texture range data. Subsequently, noises are eliminated by applying the limiting filter algorithm [51] as following: for the cloud points in each pavement profile frame, the first quartile, median, third quartile are firstly calculated. If the values of the range data are greater than 1.5 times of the third quartile value, or smaller than 1.5 times of the first quartile, they are treated as noises, whose values are replaced with the third quartile or the first quartile.

VC++ codes are developed first to import the LS-40 proprietary data into text formatted files. Afterwards, AutoLISP codes are developed to read the transformed surface texture data and the 3D surface model is re-constructed in the commercial software Autodesk^®^ (Version 2013, Autodesk Inc., San Rafae, CA, USA) [52]. The reconstructed solid 3D areal pavement surface model is exported to the commercial software ABAQUS^®^ (Version 6.13, Dassault Systèmes, Vélizy-Villacoublay, Paris, France) in sat file format. The pavement FEM mesh procedure is accomplished in ABAQUS^®^.

The influence of surface texture on friction occurs at both the macro- and micro–scale [14]. In this study, the acquired LS-40 texture data is separated into macro- and micro-texture data by using the Butterworth filter [53]. All the frequencies between 0.0008 and 0.08 cycles/m (wavelengths from 0.5 to 50 mm) are passed to isolate only the effect of macro-texture, while all the frequencies less than 0.08 cycles/m (wavelengths lower than 0.5 mm) are saved to represent the micro-texture information. Five categories of 3D areal texture parameters are calculated at the macro- and micro-texture scales and for the raw images before the separation: height parameters, spatial parameters, hybrid parameters, volume parameters, and feature parameters [40]. All the texture parameters are processed via the MountainsMap^®^ software package (Version 7.3, Digital Surf, Besançon, Bourgogne-Franche-Comté, France). The relationships between the interface friction and 3D areal texture parameters for the macro- and micro-texture data sets are discussed later.

## 6. Back-Calculation of Interface Friction Parameters

### 6.1. FEM Based Skid Resistance Simulation

In this study it is assumed that the deformations of the pavement surface are negligible as compared to that of the rubber. The 3D areal pavement surface is re-constructed as a rigid body to increase computational efficiency. The size of the pavement model is 114.3 mm × 40 mm × 8 mm. Since sharp angles can lead to computing error and convergence problems [54], the pavement element size is set as 0.5 mm. In addition, surface smoothing techniques are applied before meshing pavement surfaces. Rubber sliding on pavements is a transient dynamic behavior. During the DFT testing, the rubber block is pressing and sliding against the pavement surface. Large deformation could occur during the process, which could result in convergence problems in simulation. Therefore, the 3D linear eight-node brick element (C3D8R) [55] is selected to model the rubber slider of DFT. The size of the rubber block is 20 mm × 16 mm × 6 mm [41].

A rubber slider is a near incompressible and hyper-elastic material with viscoelasticity [56]. It is synthetic rubber as specified in the ASTM E501 specification [41,57]. The DFT rubber slider has the same compounding requirements as the standard tires used for common pavement skid-resistance testing devices, such as locked-wheel trailer and grip tester [41,57,58,59]. However, rubber manufacturers usually do not publish material’s mechanical property information. Many previous research studies have simulated the tire-pavement interaction by setting the rubber as a linear elastic material with a Poisson ratio around 0.5 [30,32,45,60]. It is also pointed out that the linear elastic material model still has the potential to demonstrate the rubber’s mechanical behavior by [61]. Hence, in this research, the linear elastic material properties are adopted from the papers by Ong et al. and Zhang et al. [45,60], in which the tire rubber material property is as same as the DFT rubber slider’s. The elastic modulus of the rubber slider is 100 MPa and the Poisson’s ratio and density of the rubber are set as 0.45 and 1200 kg/m^3^, and rubber’s viscoelastic parameter’s is adopted from the papers by Zegard et al. [61], respectively. The applied load on the slider is 11.8 N, and the sliding speeds for simulation are 20, 40 and 60 km/h. Since all the data collection was completed with very similar temperature conditions, the properties of the rubber material are considered to be constant. The rubber sliding distance in the simulation is 114.3 mm, thus the change in temperature is negligible during the friction simulation in such a short time period of testing.

**Figure 8:**
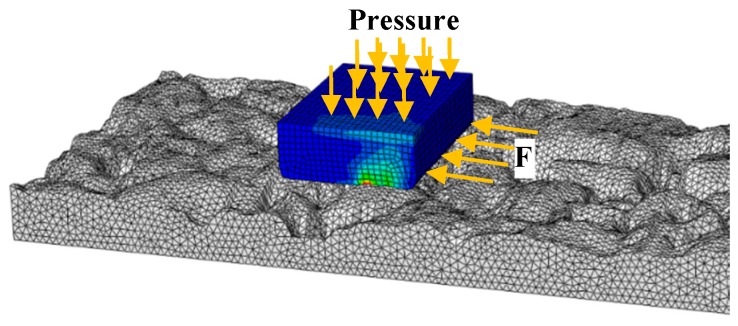
Slider-pavement interaction model.

The surface-to-surface contact algorithm incorporated in the exponential decay friction model is used to simulate the surface interaction between the rubber slider and the pavement surface morphology. The resultant tangential force is computed for the rubber slider, where velocity and loading conditions are applied. The ratio of the tangential force to the normal load is defined as the simulated skid resistance [62], as shown in Figure 8.

The water film thickness for the DFT testing is generally considered as 1 mm [41], thus the rough spots on the pavement surface with micro-rough surface characteristics are able to break-through the film of water present at the rubber-pavement interface and then form skid resistance [63]. In this research, the water’s influence on pavement friction is considered by lowering the friction parameters in the solid-solid model as a thin lubricant in the rubber-pavement interface [43]. In the FEM simulation, the exponential decay friction is “lowered to represent the introduction of a lubricant between the bodies” [54]. Such assumption was originally proposed by Oden and Martins [34] and subsequently adopted in the ABAQUS^®^ software (Version 6.13, Dassault Systèmes, Vélizy-Villacoublay, Paris, France). Additionally, the derived exponential decay friction parameters are back-calculated from the in-situ collected DFT data, and thus the influence of water on the rubber-asphalt (pavement) friction is included in the derived friction parameters.

**Figure 9:**
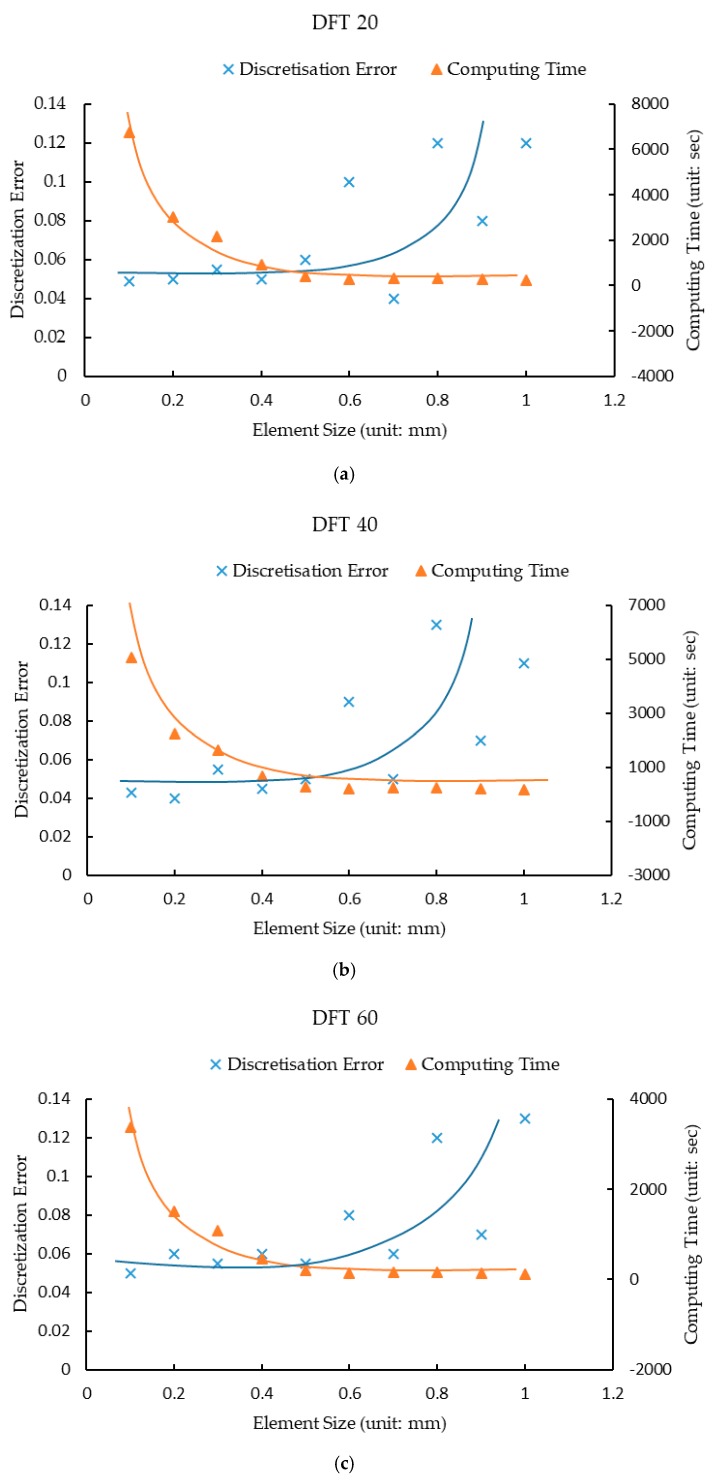
Mesh study results: (**a**) Discretization error vs. computing time at the speed of 20 km/h; (**b**) Discretization error vs. computing time at the speed of 40 km/h; (**c**) Discretization error vs. computing time at the speed of 60 km/h.

Mesh study is performed to select the appropriate element size of the slider. Assuming the exponential decay friction parameters (*μ_s_*, *μ_k_*, and α) to be 0.4, 0.35 and 0.6, the mesh study results in Figure 9 show the comparisons of the discretization errors and the computing times at the speeds of 20 km/h (DFT 20), 40 km/h (DFT 40), 60 km/h (DFT 60). It is found that the discretization errors keep less than 5% in the beginning with varying element sizes, and later significantly rises up to roughly 12% after element sizes reach 0.5 mm. The convergence could be achieved when the discretization error is less than 5% while the computing time remains low with various element sizes. As a result, considering both the numerical convergence and the computational efficiency, the rubber’s element size is selected as 0.5 mm.

### 6.2. Binary Search Back-calculation Approach

To obtain the exponential decay friction model’s parameters *μ_s_*, *μ_k_*, and α, the binary search back-calculation methid is adopted in this study. This method consistently adjusts these parameters in the FEM simulation process so that the simulated skid resistance values are approximating the in-situ measurement value within acceptable accuracy. The DFT skid resistance measurements at 20 (DFT 20), 40 (DFT 40), and 60 km/h (DFT 60) are selected as the field validation data for this method. The limits of the 95% confidence interval are fulfilled when the percentage error from the back-calculation process is less than 10% [60].

Zhou et al. [33] recommended that the static friction coefficient *μ_s_* and the kinetic friction coefficient *μ_k_* to be 0.85 and 0.70 respectively, and the decay coefficient α ranging from 0 to 1 under dry conditions. Since the friction model in this paper incorporates the influence of water lubrication, *μ_s_*, *μ_k_*, and α should fall within the ranges of [0, 0.85], [0, 0.7], and [0, 1] [38].

Using the calculation of *μ_k_* as an example, the binary search back-calculation method is composed of the following steps [64]:

(1) Calculate the midpoint value *σ*_0_ from the initial range interval [0, 0.85];
(2) Input *σ*_0_ into the FEM simulation process and obtain the simulated skid number $SN_{\sigma_{0}}$;
(3) Compare the $SN_{\sigma_{0}}$. with the in-situ skid number $SN_{in-situ}$. If the percentage error $\left(SN_{\sigma_{0}}-SN_{in-situ}\right)/\left(SN_{in-situ}\right)$ is less than 10%, the convergence is satisfied and the iteration process stops. Otherwise the iteration process continues and proceeds to step 4;
(4) Calculate the new midpoint *σ*_1_ from the subinterval [0, *σ*_0_]. If the difference from step 3 is positive or [*σ*_0_, 0.85] if the difference is negative, feed it into the FEM simulation process for simulated skid resistance $SN_{\sigma_{1}}$, and check whether the differences are within desired accuracy range. Repeat the process until the percentage error is less than 10%.

According to the exponential decay friction formula, *μ_k_* is the main factor affecting the interface friction at high speed, *μ_s_* dominantly affects the interface friction at low speed, the decay coefficient α represents the friction coefficient’s sliding velocity dependency property. Therefore, it is computationally effective to firstly back-calculate *μ_k_* at the speed of 60 km/h. Subsequently, keeping *μ_k_* constant, back-calculate *μ_s_* at the speed of 20 km/h. Finally, keeping *μ_k_*, *μ_s_* constant, back-calculate α at the speed of 40 km/h, following the back-calculation process described above until the percentage error is less than 10%. For each data set, the back-calculation process is applied to obtain the appropriate exponential decay friction parameters via a loop computing process until the computed skid resistance best fit the in-situ DFT measurements. Table 2 provides one example of the back-calculated exponential decay friction model’s parameters.

### 6.3. Validation of FEM Simulation Results

**Figure 10:**
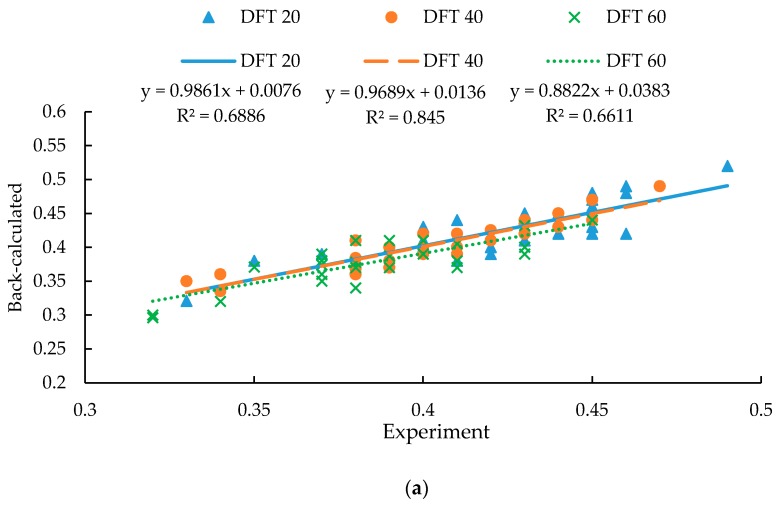

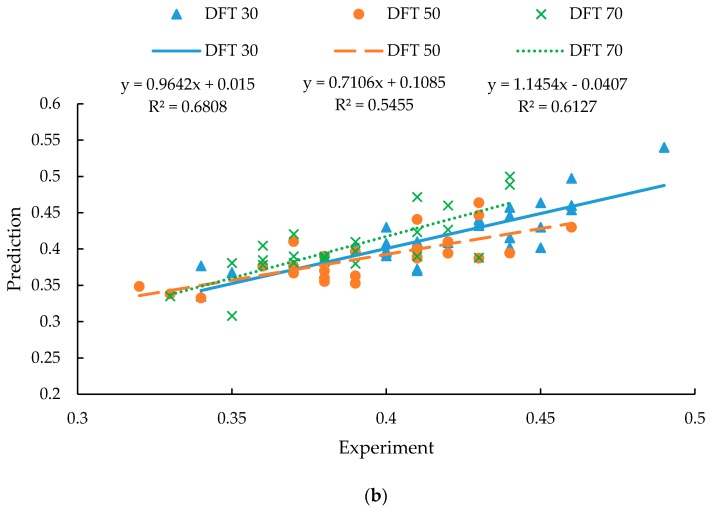
Validation of the back-calculated results: (**a**) Back-calculated results vs. experimental data; (**b**) Predicted results vs. experimental data.

The DFT measurements at the speed of 20 km/h (DFT 20), 40 km/h (DFT 40), 60 km/h (DFT 60) are then used to back-calculate the exponential decay friction model parameters. As shown in Figure 10a, the R-squared values are 0.68 to 0.85 between the simulated and DFT measured skid resistance. To further validate the back-calculation method, the skid resistance at the speed of 30 km/h (DFT 30), 50 km/h (DFT 50), 70 km/h (DFT 70), which are not used for the back-calculation process, are simulated for verification. As shown in Figure 10b, the R-squared value varied from 0.55 to 0.68, indicating that the derived exponential decay friction model parameters are satisfactory for FEM simulation.

## 7. Friction Prediction Models Based on Surface Texture Parameters

### 7.1. Model Development

Since the FEM process is time consuming and computationally extensive, it is desired to develop friction prediction models using in-situ 3D texture data sets and parameters. The detailed definitions of the 3D texture indicators at the macro- and micro-scales can be found in Table 3. These parameters have been widely used to characterize surface texture properties. The descriptive statistics of 3D areal pavement texture parameters are summarized in Table 4, which includes statistics such as average, maximum, minimum, standard deviation.

Cross-correlation analysis in previous study [16] is conducted to reveal the correlation among the 3D macro- and micro-texture indicators within each category. It has been demonstrated that a high level of correlation exists within the macro- and micro-texture indicators. In order to enable accurate mapping of the texture indicators to the friction parameters, it is important to reduce the dimensionality of the identified 3D texture indicators and then develop a multivariate regression model for friction prediction.

In this paper, principal component analysis (PCA) [65,66], a statistical procedure that uses an orthogonal transformation to convert a set of observations of possibly correlated variables into a set of values of linearly uncorrelated variables called principal components, is used for dimension reduction. The basic equation is defined as below:

(2) $$A_{m\times n}=Q_{m\times n}\times B_{m\times n}$$

where, *A* is the PC vectors matrix; *B* is the original variable matrix; *Q* is weighting coefficients matrix to establish the linear relationship between *A* and *B*; *m* is the number of variables in original matrix; *n* is the number of experiments/observations.

After the variables transformation, friction prediction models can be developed based on the significant PC vectors, as shown in Equation (3):

(3) $$\mathrm{Fction}\;\mathrm{parameter}=a^{*}+\sum_{1}^{n}P_{i}^{*}\times b_{i}^{*}$$

where, $a^{*}$ is estimated coefficient for intercept; $P_{i}^{*}$ is the PC vector; $b_{i}^{*}$ is the corresponding coefficient for the PC vector.

### 7.2. Model Verification and Discussion

The derived parameters from the exponential decay friction model and the 3D texture indicators are used as the dependent variables and explanatory variables for the PCA regression process. 27 pairs of data sets are randomly selected to develop the regression model and 9 data sets are used to validate the model.

The PCA regression results are summarized in Table 5. The *p*-values for the PC vectors are all smaller than 0.01, implying that the generated principal components are significant to the friction parameters. The adjusted R square value of the regression model for *μ_k_*, *μ_s_* and α are 0.9456, 0.8276, 0.7215, respectively, demonstrating that the friction parameters can be well explained by the principal component vectors after eliminating the multicollinearity.

To illustrate the influence of texture indicators on exponential decay friction parameters, the selected significant PC vectors are transformed back into the combinations of original texture indicators as shown in Table 6 so that the friction parameters can be directly predicted as shown in Equation (4):

(4) $$\mathrm{Friction}\;\mathrm{parameter}=a+\sum_{1}^{n}T\times b_{i}$$

where, a is estimated coefficient for intercept; T is the texture indicators; $b_{i}$ is the corresponding coefficient for texture indicators.

It can be concluded from Table 6 that texture indicators comprehensively influence the exponential decay friction parameters. Macro-texture and micro-texture commonly dominate the $\mu_{s}$, while micro-texture dominate the $\mu_{k}$ as well as the macro-texture dominate the α. However, different texture indicator plays different roles on the friction parameters. For example, Sq dominate the $\mu_{s}$, $\mu_{k}$, and α, while Ssk significantly determine $\mu_{k}$ at micro-level but fail to dominate the $\mu_{s}$ and α at either micro- or macro- level. This phenomenon also implies that the individual texture indicator’s influence on the friction parameter changes when switching from macro-level to micro-level.

**Figure 11:**
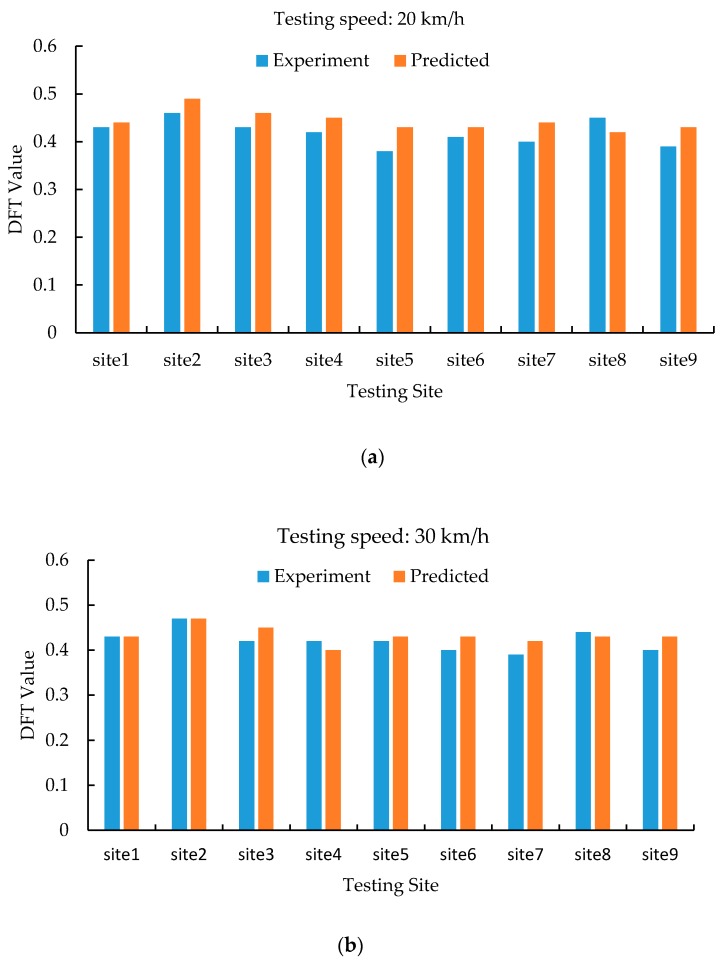

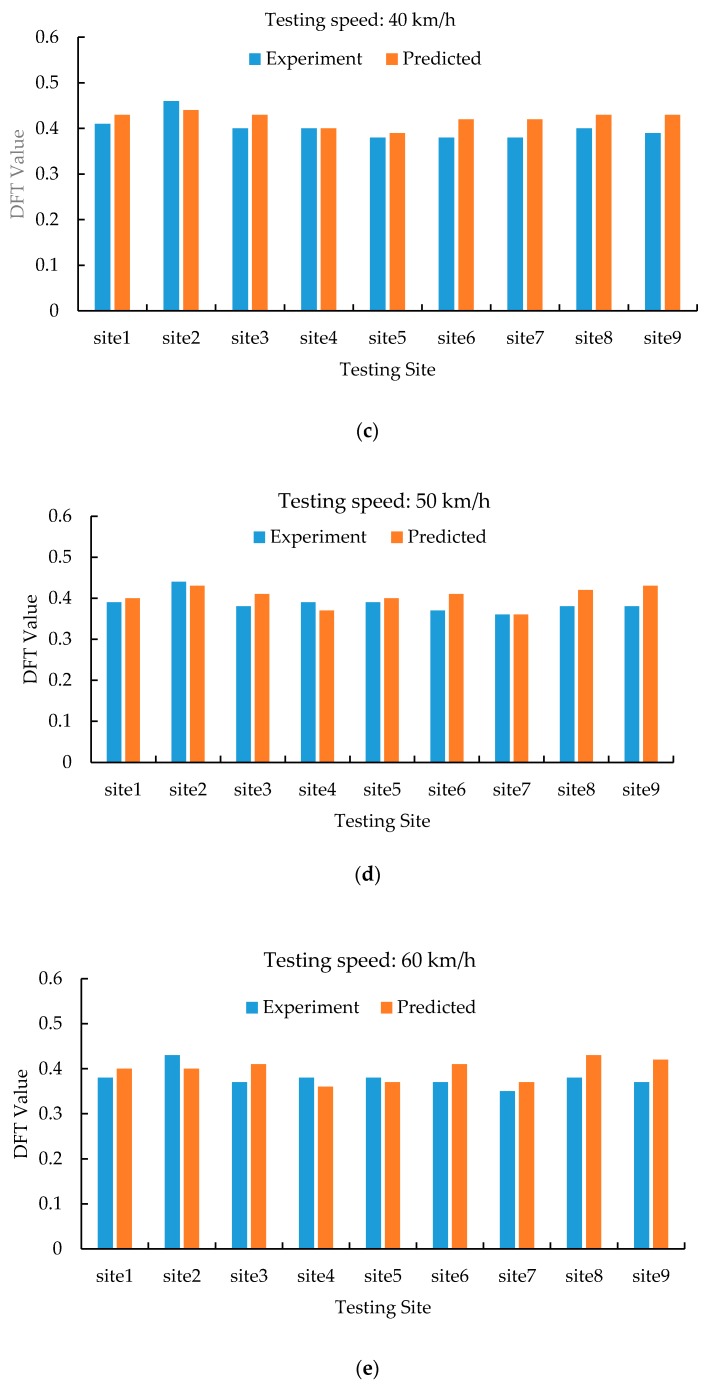

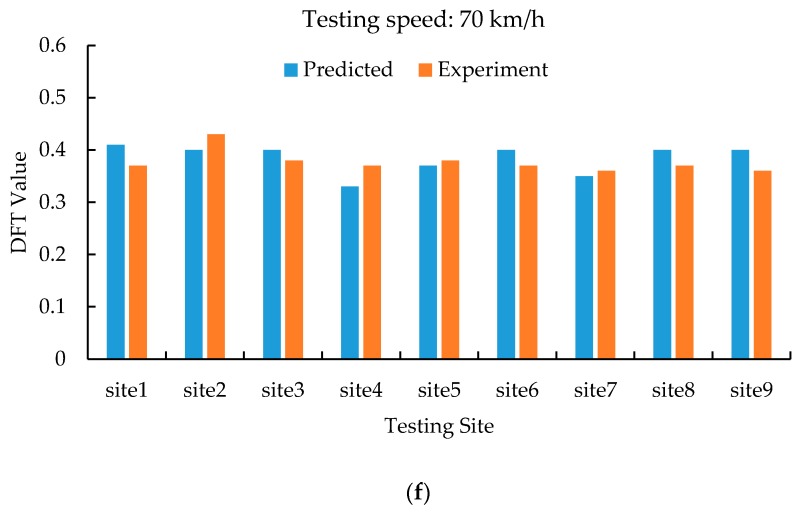
Model validation and comparisons: (**a**) Predicted results vs. experimental data at the speed of 20 km/h; (**b**) Predicted results vs. experimental data at the speed of 30 km/h; (**c**) Predicted results vs. experimental data at the speed of 40 km/h; (**d**) Predicted results vs. experimental data at the speed of 50 km/h; (**e**) Predicted results vs. experimental data at the speed of 60 km/h; (**f**) Predicted results vs. experimental data at the speed of 70 km/h.

The remaining data sets are used to compare the predicted values with actual DFT measurements, as shown in Figure 11. The differences between the predicted results and experimental data are mostly under 10%, which can be demonstrated by the largely overlapping of experimental and predicted values.

The predicted skid resistance has the similar trend with the in-situ skid resistance measured by the DFT at various testing speeds. It can be concluded that good agreements exist between the skid resistance predicted from the FE model in this research and the measurement results. This indicates that the proposed methodology can be used to predict skid resistance in terms of simulating the DFT with acceptable accuracy.

## 8. Conclusions

This study proposed a framework to quantify the relationship between texture characteristics and the interface friction coefficient and to prepare the friction inputs for the 3D based rubber pavement interaction simulation using testing data from the LTPP SPS-10 WMA testing site in Oklahoma. Comparing to the previous study [9,22], this research considers pavement texture as a significant factor for the skid resistance prediction at both macro- and micro- level. In particular, the following analyses are conducted, which could help researchers better investigate the rubber pavement interaction mechanism and aid road agencies making better pavement maintenance decisions:

- A rubber areal pavement interaction FEM model is established to determine the rubber pavement interface friction by re-constructing 3D areal pavement model from high resolution surface texture data;
- The binary search back-calculation method is used to derive the rubber pavement interface friction parameters so that the simulated skid resistance fits with the in-situ skid resistance data at a desired accuracy level;
- PCA regression models are developed to correlate interface friction parameters and the 3D areal pavement texture characteristics, which can be used to prepare the inputs of friction parameters for FEM simulation.

## Figures and Tables

**Table 1:** Mixture Design for the LTPP SPS-10 Test Sections.

Section ID	Binder	Comment	Aggregate Combination
E1	PG 70-28	HMA with RAP + RAS	1
E2	PG 70-28	WMA foaming with RAP + RAS	1
E3	PG 70-28	WMA chemical with RAP + RAS	1
E4	PG 64-22	WMA chemical with RAP + RAS	1
E5	PG 58-28	WMA chemical with RAP + RAS	1
E6	PG 70-28	WMA stone mix with mineral filler	2
C1–C6	PG 70-28	HMA with RAP	3

Note: E1–E6 represent the experimental treatment sections; C1–C6 represent the control sections; aggregate combination 1 incorporates 38% 5/8 chips + 35% stone stand + 12% sand + 12% RAP + 3% RAS; aggregate combination 2 incorporates 90% 5/8 chips + 10 mineral filler; aggregate combination 3 incorporates 34% 5/8 chips + 13% screens + 30% stone sand + 13% sand + 10% RAP.

**Table 2:** Binary search back-calculation example results.

No.	*μ_s_*	*μ_k_*	α	No.	*μ_s_*	*μ_k_*	α
1	0.4	0.35	0.5	15	0.4	0.36	0.2
2	0.4	0.35	0.5	16	0.4	0.36	0.2
3	0.4	0.36	0.2	17	0.4	0.36	0.2
4	0.4	0.39	0.6s	18	0.4	0.35	0.6
5	0.5	0.31	0.6	19	0.4	0.39	0.6
6	0.5	0.23	0.4	20	0.5	0.35	0.6
7	0.5	0.35	0.6	21	0.5	0.4	0.2
8	0.4	0.35	0.6	22	0.5	0.36	0.2
9	0.4	0.33	0.6	23	0.5	0.34	0.2
10	0.4	0.34	0.6	24	0.4	0.37	0.6
11	0.4	0.34	0.6	25	0.5	0.4	0.2
12	0.4	0.35	0.6	26	0.5	0.3	0.2
13	0.4	0.35	0.6	27	0.4	0.37	0.6
14	0.4	0.35	0.6				

**Table 3:** 3D areal pavement texture parameters definition. Data from [67,68,69,70,71].

Texture Parameter	Category	Definition	Unit
Sq	Height parameters	Root-mean-square height	mm
Ssk		Skewness	
Sp		Maximum peak height	mm
Sv		Maximum pit height	mm
Sz		Maximum height	mm
Sa		Arithmetic mean height	mm
F_dmax_		Maximum depth of surface furrows in the height parameters	mm
F_dmean_		Mean depth of surface furrows in the height parameters	mm
F_den_		Mean density of surface furrows in the height parameters	cm/cm^2^
Sal	Spatial parameters	Autocorrelation length	mm
Str		Texture-aspect ratio	
Sdq	Hybrid parameters	Root-mean-square gradient	
Sdr		Developed interfacial area ratio	%
Vm	Volume parameters	Material volume	mm^3^/mm^2^
Vv		Void volume	mm^3^/mm^2^
Vmp		Peak material volume	mm^3^/mm^2^
Vmc		Core material volume	mm^3^/mm^2^
Vvc		Core void volume	mm^3^/mm^2^
Vvv		Pit void volume	mm^3^/mm^2^
Sk		Core roughness depth	mm
Spk		Reduced summit height	mm
Svk		Reduced valley depth	mm
Spc		Arithmetic mean peak curvature	1/mm
S10z		Ten point height	mm
S5p		Five point peak height	mm
S5v		Five point pit height	mm
Shv		Mean hill volume	mm^3^
Sa2	Functional parameters	Areas below the material ratio curve	mm^3^/mm^2^
Sr1		Upper bearing area	%
Sr2		Lower bearing area	%
Threshold	Islands parameters	The threshold value to estimate the bumps contained in the height parameters	mm
Mean Volume		Mean volume of the islands	mm^3^
Mean Height/Surface ratio		Mean ratio of the height to surface of the islands	mm/mm^2^
Mean Area	Motifs parameters	Mean area of the motifs	mm
Temperature	Pavement surface temperature		°C

**Table 4:** Statistics of pavement 3D texture parameters.

	Statistical Results	Average		Maximum		Minimum		Standard Deviation	
Parameters		Macro	Micro	Macro	Micro	Macro	Micro	Macro	Micro
Sq		0.37	0.02	0.87	0.04	0.22	0.02	0.17	0.01
Ssk		−1.66	−0.33	0.02	0.02	−2.54	−0.64	0.55	0.13
Sp		1.77	0.42	4.93	0.84	0.89	0.21	0.95	0.16
Sv		2.81	0.51	3.72	0.96	1.88	0.24	0.49	0.18
Sz		4.59	0.93	8.57	1.69	3.22	0.47	1.21	0.32
Sa		0.26	0.02	0.67	0.02	0.15	0.01	0.14	0.002
F_dmax_		3.05	0.35	4.61	0.62	2.01	0.20	0.62	0.12
F_den_		11.64	18.80	12.93	19.03	8.66	18.61	1.20	0.11
Sal		2.90	0.19	4.49	0.19	2.04	0.18	0.49	0.001
Str		0.72	0.20	0.93	0.29	0.51	0.005	0.10	0.08
Sdq		0.63	0.23	1.27	0.47	0.44	0.17	0.20	0.07
Sdr		15.41	2.52	40.89	7.11	8.28	1.41	7.84	1.31
Vm		0.01	0.002	0.03	0.003	0.01	0.002	0.006	0.002
Vv		0.37	0.03	0.99	0.03	0.21	0.02	0.20	0.003
Vmp		0.01	0.002	0.03	0.002	0.01	0.007	0.01	0.004
Vmc		0.26	0.01	0.76	0.02	0.14	0.01	0.16	0.002
Vvc		0.30	0.02	0.86	0.03	0.17	0.02	0.18	0.001
Vvv		0.07	0.003	0.15	0.01	0.04	0.002	0.03	0.003
Sk		0.63	0.04	1.92	0.05	0.36	0.04	0.39	0.003
Spk		0.28	0.03	0.67	0.07	0.15	0.02	0.12	0.01
Svk		0.70	0.04	1.45	0.07	0.39	0.02	0.26	0.01
Spc		9.15	10.45	45.01	47.14	5.43	4.30	8.74	11.06
S10z		3.19	0.68	6.29	1.38	2.18	0.41	0.93	0.22
S5p		1.07	0.32	3.20	0.67	0.35	0.19	0.63	0.11
S5v		2.12	0.37	3.09	0.71	1.40	0.21	0.42	0.11
Shv		1.81	0.03	6.26	0.12	0.70	0.01	1.40	0.02
Sa2		0.0595	0.0022	0.1303	0.0046	0.0316	0.0012	0.0221	0.0007
Sr1		8.64	11.4427	11.28	13.999	7.25	10.429	1.01	0.7643
Sr2		82.54	88.2025	84.97	89.333	81.15	87.317	1.03	0.5326
Threshold		1.6029	0.3999	4.4747	0.8204	0.862	0.2094	0.7711	0.1268
Mean Volume		10135	890.9	22357	3566.1	0.32	0.0022	7925.8	1131.04
Mean Height/Surface ratio		1.2094	1.0105	13.5843	13.132	0.0002	2.929	2.9249	2.5314
Mean Area		45.906	6.5764	106.636	14.94	25.104	2.4568	18.215	3.3
Temperature		15.8	15.8	18.9	18.9	11.8	11.8	3.64	3.64

**Table 5:** PCA Regression results for the exponential decay friction parameters.

Component		*μ_s_*		*μ_k_*		α	
		Coefficient	*p*-Value	Coefficient	*p*-Value	Coefficient	*p*-Value
Intercept		4.503 × 10^−01^	***	3.504 × 10^−01^	***	4.519 × 10^−01^	***
1		0.1022 × 10^−01^	**	−0.03165 × 10^−01^	***	−0.1298 × 10^−01^	*
2		0.2557 × 10^−01^	***	0.04643 × 10^−01^	**	−0.4774 × 10^−01^	*
3		−0.1781 × 10^−01^	**	0.2217 × 10^−01^	***	0.7817 × 10^−01^	*
4		0.4148 × 10^−01^	**	0.1323 × 10^−01^	*	−5.040 × 10^−01^	*
5		0.7041 × 10^−01^	***	0.2229 × 10^−01^	*	41.24 × 10^−01^	**
6		−1.349 × 10^−01^	***	−7.055 × 10^−01^	***	−63.63 × 10^−01^	**
7		2.895 × 10^−01^	***	12.98 × 10^−01^	**	228.3 × 10^−01^	**
8		−4.715 × 10^−01^	***	−17.40 × 10^−01^	*	−767.0 × 10^−01^	**
9		−2.459 × 10^−01^	**	−22.91 × 10^−01^	**	−1786.0 × 10^−01^	***
10		3.975 × 10^−01^	***				
11		−13.93 × 10^−01^	***				
12		−187.3 × 10^−01^	***				
13		114.0 × 10^−01^	***				
14		−1120.0 × 10^−01^	***				
15		699.5 × 10^−01^	***				
16		−313.8 × 10^−01^	***				
17		1933.0 × 10^−01^	***				
Validation Results	*p*-value	***		***		***	
	R2	9.812 × 10^−01^		8.873 × 10^−01^		8.179 × 10^−01^	
	Adjusted R2	9.456 × 10^−01^		8.276 × 10^−01^		7.215 × 10^−01^	
	RSE	0.1027 × 10^−01^		0.1379 × 10^−01^		0.9858 × 10^−01^	
No. of Samples		27					

Notes: Significant values: *, *p* < 0.5 × 10^−01^; **, *p* < 0.1 × 10^−01^; ***, *p* < 0.01 × 10^−01^. The more *p*-value with star marks, the higher significance exists between the principal component and exponential decay friction parameters.

**Table 6:** PCA Regression coefficients for texture characteristics.

Item	*μ_s_*		*μ_k_*	α	Item	*μ_s_*		*μ_k_*	α
	Coefficient		Coefficient	Coefficient		Coefficient		Coefficient	Coefficient
	Macro	Micro	Micro	Macro		Macro	Micro	Micro	Macro
Intercept	0.0029		−0.0336	0.7926	Vvc	−0.0250		−0.9959	0.0187
Sq		1.2279	−0.0786	0.0217	Vvv		9.2543	−0.6802	0.2615
Ssk			0.0076		Sk	−0.0103			0.0106
Sp			0.0012		Spk		0.3215		−0.1864
Sv	0.0328				Svk		0.3706	−0.0115	0.0275
Sz			0.0011		Spc	−0.0008	−0.0004		−0.0002
Sa			−0.7194	0.0345	S10z				−0.0277
F_dmax_	0.0187				S5p		0.0099	0.0023	
F_dmean_	−0.0152		−0.2727	0.0083	S5v	0.0253			
F_den_			0.0197		Shv				−0.0151
Sal		4.5669	−0.67	−6.1146	Sa2		7.2769	−0.3313	
Str			−0.0280		Sr1		0.0121	−0.0007	
Sdq	0.0152	0.0349		0.054	Sr2			0.0025	
Sdr	0.0004	0.0023	0.0001	0.0015	Threshold			0.0012	
Vm		4.3611		−3.275	Mean Height/Surface ratio	−0.0017		0.0003	−0.0002
Vv	−0.0182		−0.6727	0.0195	Mean Area				−0.0934
Vmp		4.3611		−3.275	Temperature			0.0004	−0.0022
Vmc	−0.0226		−1.7592	0.0317					
